# Correction

**DOI:** 10.1111/cas.15797

**Published:** 2023-04-04

In an article^1^ titled “Triptolide abrogates oncogene FIP1L1‐PDGFRα addiction and induces apoptosis in hypereosinophilic syndrome” by Yanli Jin, Qi Chen, Zhongzheng Lu, Bo Chen, Jingxuan Pan, there were errors in Figure 5 and in the legends for Figures 1, 3, 4 and 5.

The correct Figure 5 and its legend are shown below:

**Figure:**
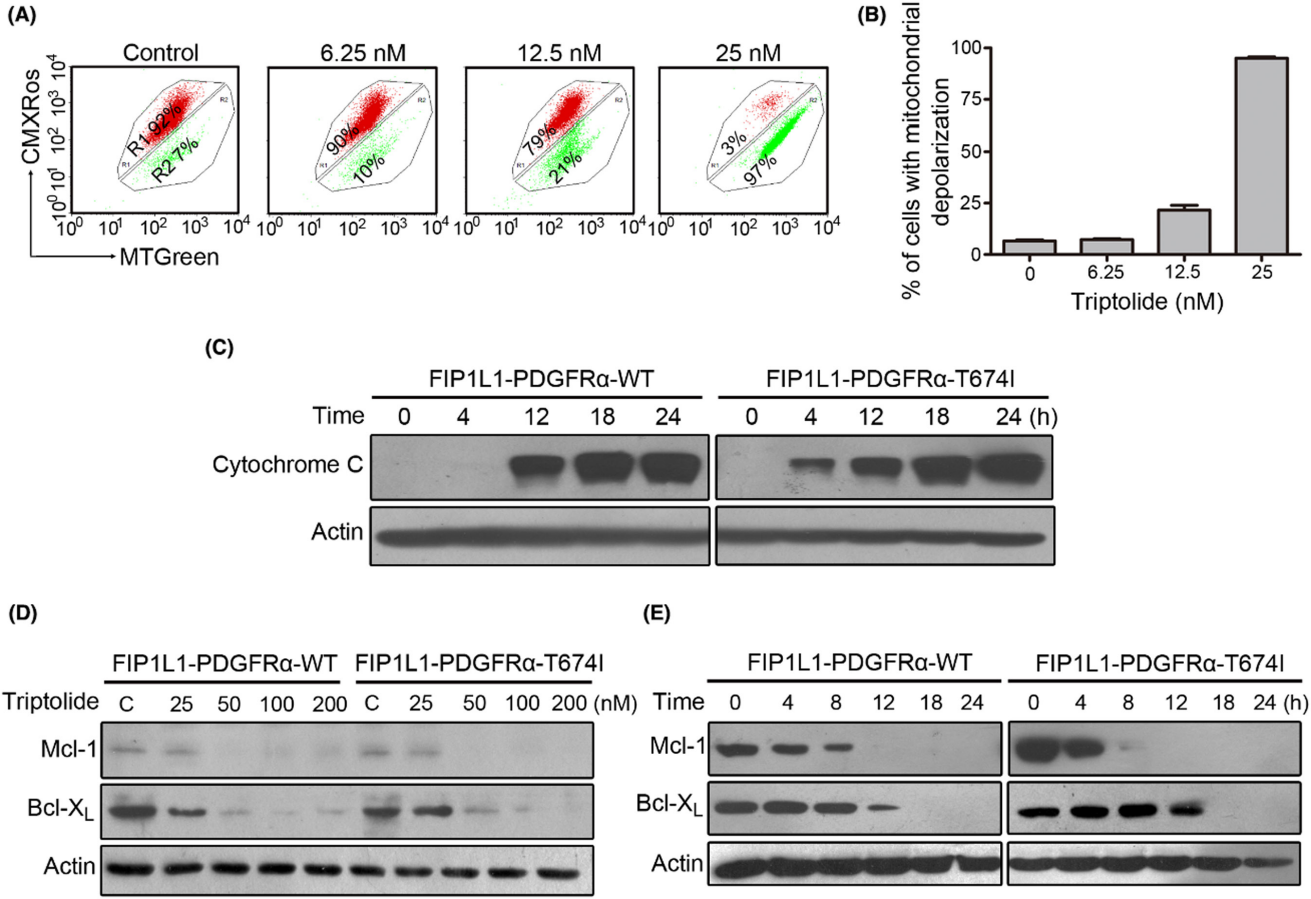
**FIGURE 5** Triptolide leads to mitochondrial damage, cytochrome c release, and alteration in apoptosis‐related proteins. (A, B) EOL‐1 cells were treated with triptolide for 24 h, then stained with CMXRos and MTGreen, and mitochondrial transmembrane potential (Δ*Ψ*m) was immediately analyzed by flow cytometry. R1 in the histograms (A) represents the population of cells with intact mitochondria; and R2, the population with loss of mitochondrial potential. The histograms are representative results from three independent experiments (A). Values represent mean ± SE (B). (C) After exposure to triptolide for 24 h, BaF3 cells expressing WT or T674I FIP1L1‐PDGFRα were harvested for cytosolic fraction extraction to detect cytochrome c in the cytosol. (D, E) BaF3 cells expressing WT or T674I FIP1L1‐PDGFRα were exposed to increasing concentrations of triptolide for 24 h (D) or 100 nM triptolide for various times (E), the apoptosis‐related proteins were detected by Western blot analysis. The immunoblot bands against Actin in Figure 5D, Figure 1B, Figure 3A and Figure 4D were from the same set of lysates isolated from the indicated cells treated with different concentrations of triptolide. We did the immunoblotting experiments at the same time, and the data were just arranged into different panels while narrating story. Thus, Figure 5D, Figure 1B, Figure 3A and Figure 4D shared the inner standard. The immunoblot bands against Actin in Figure 5E, Figure 1C and Figure 3B were from the same set of lysates isolated from the indicated cells treated with triptolide for various durations. We did the immunoblotting experiments at the same time, and the data were just arranged into different panels while narrating story. Thus, Figure 5E, Figure 1C and Figure 3B shared the inner standard.

The correct legends for Figures 1, 3 and 4 are shown below:

**FIGURE 1** Triptolide potently inhibits the expression and activity of platelet‐derived growth factor receptor alpha (PDGFRα), counteracting the growth of neoplastic cells expressing mutant PDGFRα. (A) Human hypereosinophilic syndrome (HES) EOL‐1 cells were incubated for 24 h in the absence or presence of increasing concentrations of triptolide. Phosphorylated and total levels of PDGFRα were detected by Western blot analysis. C denotes control treatment with medium containing DMSO (<0.1%). The immunoblot bands against Actin in Figure 1A and Figure 4C were from the same set of lysates isolated from the EOL‐1 cells treated with different concentrations of triptolide. We did the immunoblotting experiments at the same time, and the data were just arranged into different panels while narrating story. Thus, Figure 1A and Figure 4C shared the inner standard. (B, C) The dose‐ and time‐dependent decrease of phosphorylated RNA pol II and PDGFRα in BaF3 cells expressing WT or T674I FIP1L1‐PDGFRα. The indicated cells were exposed to various concentrations of triptolide for 24 h (B) or 100 nM triptolide for various time periods (C). PDGFRα and its phosphorylation were measured by Western blot analysis. The immunoblot bands against Actin in Figure 1B, Figure 3A, Figure 4D and Figure 5D were from the same set of lysates isolated from the indicated cells treated with different concentrations of triptolide. We did the immunoblotting experiments at the same time, and the data were just arranged into different panels while narrating story. Thus, Figure 1B, Figure 3A, Figure 4D and Figure 5D shared the inner standard. The immunoblot bands against Actin in Figure 1C, Figure 3B and Figure 5E were from the same set of lysates isolated from the indicated cells treated with triptolide for various durations. We did the immunoblotting experiments at the same time, and the data were just arranged into different panels while narrating story. Thus, Figure 1C, Figure 3B and Figure 5E shared the inner standard. (D) Triptolide decreases mRNA levels of PDGFRα. Semi‐quantitative RT‐PCR analysis of mRNA levels of PDGFRα in HES cells which were treated with triptolide at the indicated concentrations and durations. Ethidium bromide–stained gel of the PCR products. (E) Triptolide accelerated the turnover of PDGFRα. EOL‐1 and BaF3 cells expressing WT or T674I FIP1L1‐PDGFRα were incubated in the absence of presence of 12.5 nM (for EOL‐1 cells) or 100 nM (for BaF3 cells) triptolide for 6 h, followed by treatment with 5 μg/mL of cycloheximide (CHX) for different durations. Western blot was employed to detect PDGFRα expression (Left panel). The immunoblots were quantified by densitometry. Levels of phosphorylation were normalized to the levels of relevant total protein or β‐actin, and then normalized relative controls incubated with DMSO containing medium. The graphs (right panel) were one representative result from three independent experiments. (F) Seventy‐two hour in vitro treatment by triptolide, followed by MTS assay. IC50 values were the means of three independent experiments Error bars indicate SE.

**FIGURE 3** Triptolide decreases the phosphorylation of the platelet‐derived growth factor receptor alpha (PDGFRα) downstream signal molecules Stat3, Erk1/2, and Akt. (A) After 24 h treatment with increasing concentrations of triptolide, BaF3 cells expressing WT or T674I FIP1L1‐PDGFRα were analyzed by Western blot analysis. The immunoblot bands against Actin in Figure 3A, Figure 1B, Figure 4D and Figure 5D were from the same set of lysates isolated from the indicated cells treated with different concentrations of triptolide. We did the immunoblotting experiments at the same time, and the data were just arranged into different panels while narrating story. Thus, Figure 3A, Figure 1B, Figure 4D and Figure 5D shared the inner standard. (B) BaF3 cells expressing WT or T674I FIP1L1‐PDGFRα were exposed to triptolide at 100 nM for ∼24 h, then the expression and phosphorylation of Stat3, Erk1/2, and Akt were analyzed by Western blot analysis. The immunoblot bands against Actin in Figure 3B, Figure 1C and Figure 5E were from the same set of lysates isolated from the indicated cells treated with triptolide for various durations. We did the immunoblotting experiments at the same time, and the data were just arranged into different panels while narrating story. Thus, Figure 3B, Figure 1C and Figure 5E shared the inner standard.

**FIGURE 4** Triptolide induces apoptosis in cells expressing FIP1L1–platelet‐derived growth factor receptor alpha (PDGFRα). EOL‐1 or BaF3 cells expressing WT or T674I FIP1L1‐PDGFRα were treated with triptolide at increasing concentrations for 24 h, apoptosis assay with flow cytometry by PI/annexin V‐FITC (A) or 7AAD/annexin V‐PE (B) staining; or Western blot analysis of the indicated proteins (C, D). The immunoblot bands against Actin in Figure 4C and Figure 1A were from the same set of lysates isolated from the EOL‐1 cells treated with different concentrations of triptolide. We did the immunoblotting experiments at the same time, and the data were just arranged into different panels while narrating story. Thus, Figure 4C and Figure 1A shared the inner standard. The immunoblot bands against Actin in Figure 4D, Figure 1B, Figure 3A and Figure 5D were from the same set of lysates isolated from the indicated cells treated with different concentrations of triptolide. We did the immunoblotting experiments at the same time, and the data were just arranged into different panels while narrating story Thus, Figure 4D, Figure 1B, Figure 3A and Figure 5D shared the inner standard. (E) Time‐dependent analysis of PARP cleavage in BaF3 cells expressing WT or T674I FIP1L1‐PDGFRα after treatment with 100 nM triptolide for various times. (F) Silencing PDGFRα induces cell death in HES cells. EOL‐1 cells or BaF3 cells expressing WT‐FIP1L1‐PDGFRα were transfected with siRNA duplexes against either human PDGFRα or Luciferase GL2 (mock siRNA) according to the procedure described in “Materials and Methods”. Twenty‐four or 48 h after siRNA transfection, the cells were evaluated by Western blot analysis and cell death assay (Trypan blue exclusion, bottom, the columns corresponded to the lanes). ***P < 0.001, Student's *t*‐test based on three independent experiments.

The authors apologize for the errors.
